# Surface Analysis of a Universal Resin Composite and Effect of Preheating on its Physicochemical Properties

**DOI:** 10.1590/0103-6440202305411

**Published:** 2023-10-27

**Authors:** Marcus Vinícyus Manoel da Silva, João Marcos Nascimento Batista, May Anny Alves Fraga, Américo Bortolazzo Correr, Edson Alves de Campos, Saulo Geraldeli, Mário Alexandre Coelho Sinhoreti

**Affiliations:** 1 Department of Restorative Dentistry, Dental Materials Division, Piracicaba Dental School, University of Campinas, Piracicaba, SP, Brazil; 2 Department of Restorative Dentistry, Araraquara Dental School, Sao Paulo State University, Araraquara, SP, Brazil; 3 Division of Biomedical Materials, School of Dental Medicine, East Carolina University, Greenville, NC, USA.

**Keywords:** Resin composite, nanoparticles, dental materials, properties, heating

## Abstract

This study was aimed at analyzing the surface properties of a universal resin composite and evaluating the effect of preheating on its physicochemical properties. Two commercial resin composites were used under two conditions: Filtek Universal Restorative (UR); UR preheated (URH); Filtek Supreme (FS) and FS preheated (FSH). The film thickness (FT) test (n = 10) was done using two glass slabs under compression. Flexural strength (FLS) and modulus (FLM) were evaluated using a three-point flexion test (n = 10). Polymerization shrinkage stress (PSS) was evaluated in a universal testing machine (n = 5). Gap width (GW) between composite and mold was measured in internally polished metallic molds (n = 10). The degree of conversion (DC) was evaluated by Fourier Transform Infrared spectroscopy (n = 3). The morphology of the filler particles was checked by scanning electron microscope (SEM) and EDX analysis. Surface gloss (SG) and surface roughness (SR) were evaluated before and after mechanical brushing (n = 10). The outcomes were submitted to 2-way ANOVA and Tukey's test (α = 0.05). Lower mean values of FT were observed for the preheated groups when compared to the non-preheated groups. URH and FSH showed higher mean values of FLS and FLM when compared with UR and FS. No differences were observed between groups in the PSS test. The GW was higher for the UR and FS groups when compared with URH and FSH. The DC was higher for preheated resin composites when compared to the non-preheated groups. The SR of the UR composite was higher than the FS after mechanical brushing, while the SG was higher for the FS groups. In conclusion, the universal resin composite tested generally presented similar physicochemical properties compared with the nanofilled resin composite and either similar or slightly inferior surface properties. The preheating improved or maintained all properties evaluated.

## Resumo

Neste estudo avaliou-se propriedades físico-químicas e de superfície de um compósito universal pré-aquecido e comparado a um compósito convencional. Foram utilizados dois compósitos comerciais: Filtek Universal Restorative (UR); UR pré-aquecido (URH); Filtek Supreme (FS) e FS pré-aquecido (FSH). O teste de espessura de película (EP) (n = 10) foi feito usando duas placas de vidro sob compressão. A resistência à flexão (RF) e o módulo flexural (MF) foram avaliados por meio do teste de flexão de três pontos (n = 10). A tensão de contração de polimerização (TCP) foi avaliada em uma máquina de teste universal (n = 8). A largura da fenda (LF) entre o compósito e o molde foi medida em moldes metálicos polidos internamente (n = 10). O grau de conversão (GC) foi avaliado por espectroscopia de infravermelho com transformada de Fourier (n = 3). A morfologia das partículas de carga foi observada em microscopia eletrônica de varredura (MEV) e sua composição em EDX. Brilho superficial (BS) e rugosidade superficial (RS) foram avaliados antes e após escovação mecânica (n=10). Os resultados foram submetidos à ANOVA 2-fatores e as médias comparadas pelo teste de Tukey (α = 0,05). Menores valores médios de EP foram observados para os grupos pré-aquecidos quando comparados aos grupos não pré-aquecidos. URH e FSH apresentaram maiores valores médios de RF e MF quando comparados com UR e FS. Não foram observadas diferenças entre os grupos no teste TCP. A LF foi maior para os grupos UR e FS quando comparados com URH e FSH. O GC foi maior para os compósitos pré-aquecidos quando comparados aos não pré-aquecidos. A RS do compósito UR foi maior que o FS após a escovação mecânica, enquanto o BS foi maior para os grupos FS. Em conclusão, o compósito universal testado geralmente apresentou propriedades físico-químicas semelhantes em comparação ao compósito nanoparticulado e propriedades de superfície semelhantes ou ligeiramente inferiores. O pré-aquecimento melhorou ou manteve todas as propriedades avaliadas.

## Introduction

Resin composites' aesthetic, mechanical, and chemical properties have improved considerably over the years. The aesthetic properties of resin composites are fundamental for the success of the treatment, especially in terms of longevity ^1^. Color shades available for most resin composites comply with guides like Vita Classic and mimic the dental optical properties - color, translucency, opacity, opalescence, and fluorescence ^2^. The challenge among dental practitioners is the wide variation in color shades available for some marketed resin composites ^3^.

For restorations made with resin composite of multiple shades, the resin composite has to be layered in small portions to mimic the optical properties of dental tissues. This procedure requires more processing steps, more technical sensitivity by the clinician, and longer chair time ^4^. Thus, universal resin composites have been developed to minimize these procedural challenges, ensuring desirable aesthetics and adequate mechanical properties ^3^.

When compared with traditional resin composites, universal resin composites provide a limited number of shades, which seem to match and assimilate the shades of tooth structures ^4^. This color assimilation, reported as the “chameleon effect,” refers to the ability of a dental material to acquire the color of its surrounding dental tissues, a phenomenon that seems not to occur with traditional resin composites ^4^.

In addition to their aesthetic properties, concerning the success of a dental restoration, resin composites also depend on physicochemical properties such as abrasion resistance, shrinkage stress, degree of conversion, flexural strength, flexural modulus, and viscosity, all of which result from the combination of an organic matrix and filler particles ^5^. Extrinsic factors, such as irradiance and the quality of light of the light curing device, are known to provide resin composites with adequate physicochemical properties ^6^.

Preheating the resin composite is another extrinsic factor. It is known to decrease viscosity in resin composites, enabling their use as cementing agents for ceramic veneers and other indirect restorations ^7^ ^,^ ^8^. Theoretically, a preheated resin composite with content of filler particles higher than that of resin cement could reduce the incidence of fractures in ceramic restorations ^9^ and marginal degradation of the cemented area ^9^ ^,^ ^10^ and increase color stability ^11^. Preheating is also known to improve the adaptation of the resin composite into the cavities and increase its degree of conversion and, consequently, its mechanical properties ^12^.

The durability of a dental restoration using resin composites might be related to their physicochemical and surface properties. Therefore, the present study aimed to analyze the gloss and surface roughness of a universal resin composite and evaluate the effect of preheating on its film thickness, flexural strength, flexural modulus, polymerization shrinkage stress, gap formation, and degree of conversion. A morphological analysis of their filler particles was carried out through scanning electron microscopy. The null hypotheses were that (i) the physicochemical and surface properties of the universal resin composite would not significantly differ from those of the conventional composite and that (ii) preheating would have no influence on the physicochemical properties of the resin composites tested.

## Materials and methods

### Study Design

This study evaluated two commercial resin composites (Box 1) assigned to four groups: Filtek Universal Restorative (UR); Filtek Universal preheated (URH); Filtek Supreme (FS); and Filtek Supreme preheated (FSH). The dependent variables analyzed were: film thickness (FT); flexural strength (FS); flexural modulus (FM); polymerization shrinkage stress (PSS); gap width (GW); polymerization kinetics (PK); degree of conversion (DC); surface roughness (SR); surface gloss (SG); and filler particles (FP). A sample size calculation (α = 0.05 and β = 0.2) for each test performed was carried out based on a pilot study.

**Box 1:**
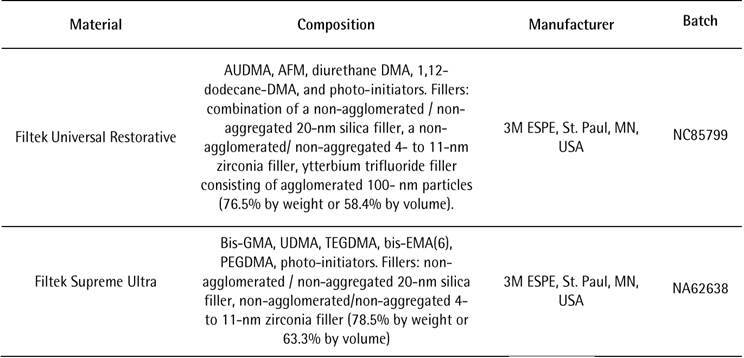
Composition, manufacturers, and batch numbers of the resin composites tested.

AUDMA - Aromatic Urethane Dimethacrylate; AFM - Addition-Fragmentation Monomer; DMA - dimethacrylate; BisGMA - bisphenol glycidyl methacrylate; UDMA - urethane dimethacrylate; TEGDMA - Triethylene glycol dimethacrylate; BisEMA - Ethoxylated bisphenol-A-diglycidyldimethacrylate; PEGDMA - Polyethylene glycol dimethacrylate.

The commercial resin composites were preheated at 69ºC ^13^ using a dental composite warmer (CalSet, AdDent Inc., Danbury, CT, USA). The temperature was checked by a digital infrared thermometer (LY-F1 Loye, São Paulo, Brazil) before each physicochemical test, except for surface roughness and surface gloss tests, where no preheating was applied.

### Film Thickness (FT)

The film thickness (n = 10) test was done in conformity with ISO 4049:2019. Two glass plates with a contact surface area of 200 mm^2^ were placed together and their total thickness was measured using a digital caliper (Mitutoyo, Tokyo, Japan). The measurement was made four times before a standardized volume of composite (0.10 mL) was inserted between them. A load of 150 N was applied for 180 s on the upper plate. The resin composite of each group was photoactivated at 1000 mW/cm^2^ (Valo Cordless, Ultradent, USA) for 40 s, following the double-time of photoactivation conforming to ISO 4049:2019 ^14^. The total thickness of the two plates was measured again four times after photoactivation. The mean value of the four final measurements was subtracted from that of the initial ones to define the film thickness.

### Flexural Strength (FLS) and Flexural Modulus (FLM)

Bar-shaped specimens (25 × 2 × 2 mm; n = 10) were fabricated in conformity to ISO 4049:2019 ^14^ using a stainless-steel mold. The resin composites were placed in the molds and light cured (Valo Cordless, Ultradent, USA) at 1000 mW/cm^2^ for 20 s with the light tip in contact with the polyester strip on the specimens’ top surface. Due to the length (25 mm) of the specimens, light-curing was performed in five overlapping irradiation cycles since the tip of the light-curing unit was 10 mm wide. The same procedure was done on the other side of each specimen. The specimens were stored in water at 37 ºC for 24 h and then submitted to the three-point (20-mm span) bending test to measure the FLS and FLM using a universal testing machine (Instron, model 4411, Canton, MA, USA) at a crosshead speed of 0.5 mm/min until failure. FLS was expressed in megapascal (MPa) and FLM in gigapascal (GPa) using the following equations:

$$FLS=\;\frac{3\;x\;L\;x\;D}{2\;x\;Wx\;h^{2}}\;\;$$

and

$$\;FLM=\;\frac{L\;x{\;D}^{3}}{4\;x\;W\;x\;h^{3}x\;d}\;x\;10^{-3}\;$$

Where L was the maximum load at failure (N), D was the distance (span) between the rods, W was the specimen’s width, h was the specimen height, and d was the crosshead displacement.

### Polymerization Shrinkage Stress (PSS)

**Figure 1:**
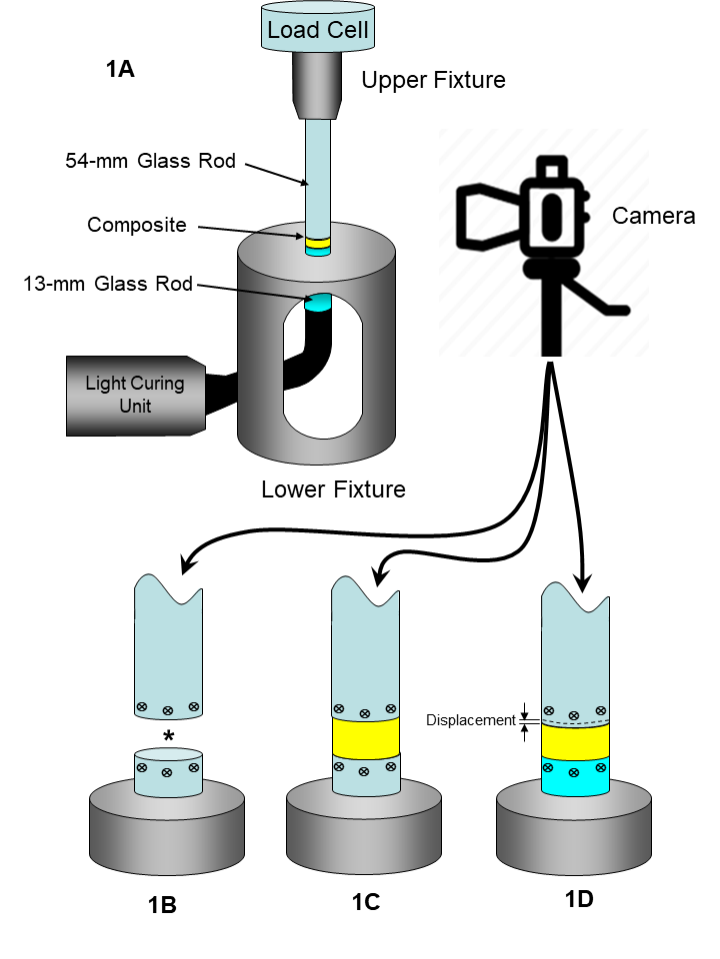
Video extensometer and its components (1A); Initial image (1B) and before placing the resin composite over the inferior glass rod (1C); after photoactivation (1D). The asterisk (*) shows the standardized distance of 1 mm between the glass rods.

The polymerization shrinkage stress (n = 5) was measured by using a universal testing machine (Instron 4411, Instron, Canton, MA, USA) ^15^. Two glass rods (radius: 2 mm) had their contact ends polished with #180-grit sandpaper (Buehler, Lake Bluff, IL, USA), etched with 10% hydrofluoric acid (Dentsply Sirona, São Paulo, Brazil), washed for 10 s under tap water, and air-dried. A single layer of silane (Monobond Plus, Ivoclar Vivadent, AG, Schaan, Liechtenstein) was applied to both end surfaces. The glass rods were then fixed to a PSS testing device (ODEME Dental Research, Luzerna, SC, Brazil) - one (54 mm in length) in the upper and the other (13 mm in length) in the lower fixture, below which the tip of the light curing unit was positioned in contact with the glass rod (Figure 1). The rods were vertically aligned and held in place, ensuring they were standardly spaced 1 mm apart.

A video extensometer was used to measure the displacement (µm) of the glass rods (Rocha et al., 2022). It consists of a video strain gauge system (Figure 1A), including a DSLR camera (Canon t3i, USA), a 100-mm macro lens (Canon, USA), and the image software that calculates the displacement (Trackmate, Fiji, ImageJ, National Institute of Health, Bethesda, MD, USA) ^16^. The camera was positioned perpendicularly to the space between the rods to calculate their displacement (µm) considering three images: before (Figure 1B) and after (Figure 1C) composite placement and after photoactivation (Figure 1D). The compliance of the strain gauge system was calculated (1.66 µm/N; C factor of 0.5) to adjust the nominal stress values.

The resin composite was placed between rods and then light cured (Valo Cordless, Ultradent, USA; 1000 mW/cm^2^). The photoactivation time was increased to 25 seconds to compensate for the 20% irradiance attenuation, which occurs as the curing light passes through the lower rod. Ten minutes after light curing, the PSS of the resin composite was measured considering the rod displacement (µm) recorded, as shown in Figure 1D. The displacement was divided by the maximum nominal strength (N) recorded on the universal testing machine, considering two compliances - 0.4 μm/N (for Class I restorations) and 3 μm/N (for Class II restorations) ^17^. A previously reported formula ^18^ was used to calculate the maximum polymerization shrinkage stress (MPa) by summing the PS_nominal_ and PS_corrected_ and dividing the maximal force by the known cross-sectional area of the glass rod.

$$Nominal\;Polymerization\;Stress\;\left({PS}_{nominal}\right)={Force}_{universal\;testing\;machine\;(N)}$$

$$Corrected\;Polymerization\;Stress\;\left({PS}_{corrected}\right)=\left[Strain\;\left(µm\right)\;x\;\frac{{Compliance}_{apparatus}}{{Compliance}_{cavity}}\right]$$

### Gap Width (GW)

The specimens (n = 8) were fabricated using an inner-polished circular brass mold (2 mm in height and 7 mm in inner diameter). The resin composites were inserted in the molds, covered with a polyester strip, and pressed with a glass slab. The upper surface of the specimens was then light cured (Valo Cordless, Ultradent, USA) for 20 s and dry-stored at 37 ºC for 24 h.

After storage, the specimens were polished with sandpaper discs - #320, #400, #600, and #1200 grit (Buehler, Lake Bluff, IL, USA) - and their top surface was metalized for the scanning electron microscopy (SEM; LEO 435 VP) analysis using the SEM software in low vacuum, at 500x magnification. The gap width was measured in four clock-face positions, corresponding hours 3, 6, 9, and 12; a mean value for each specimen was then calculated based on the arithmetic mean (µm) of the four measurements ^19^.

### Polymerization Kinetics (PK)

The polymerization kinetics (n = 3) was evaluated by Fourier-transformed near-infrared (NIR) spectroscopy (Vertex 70; Bruker Co., Ettlingen, Germany). Spectra were acquired at 4 cm^-1^ with 2 scans/s in real time on cylindrical specimens (8 mm in diameter x 0.8 mm in thickness) fabricated in rubber molds sandwiched between glass slides. Spectra were collected for 300 s. The resin composite was light-cured for 20 seconds. The degree of conversion (DC) was calculated, considering the vinyl peak (6165 cm^−1^) for methacrylate, using the equation as follows:

$$\text{DC}\left(\text{\%}\right)\text{=}\left(\text{1-}\frac{\text{6165 cm-1 cured}}{\text{6165 cm-1 uncured}}\right)\text{x 100}$$

The maximum rate of polymerization (Rp_max_) was obtained at the first derivate of the DC versus time. The Rp_max_ and the DC values were plotted to verify the vitrification point.

### Filler Particle (FP) Analysis

The morphology of the filler particles was checked by scanning electron microscope (SEM) analysis (JSM - 5600, JEOL Ltd. Tokyo, Japan). Non-polymerized resin composite (0.5 g) was centrifuged (1000 rpm) in 5 mL of acetone for 2 minutes to dissolve the resin matrix. This process was repeated three times in acetone and another three times in chloroform for further washing and full dissolution of the resin matrix. The filler particle powder was then smeared in aluminum stubs, gold-sputter coated with gold-palladium in a high vacuum (SCD 050, Bal-tec AG, Liechtenstein), and examined through SEM operating at 15 kV and 1200x magnification.

The filler particle powder was submitted to energy dispersive X-ray (EDX) analysis to identify and quantify its chemical composition. For this, the filler particle powder was smeared in acrylic resin stubs, carbon coated (Denton Vacuum Desk II Sputtering, Denton Vacuum, Cherry Hill, NJ, USA), and then submitted to SEM/EDX (JSM - 5600, JEOL Ltd. Tokyo, Japan/ Vantage 1.4, Noran Instruments, Tokyo, Japan). EDS analyses were carried out at 10 kV and a working distance of 20 mm; each specimen was three-line scanned for 100 s.

### Surface Roughness (SR) and Surface Gloss (SG)

Disk-shaped specimens (n = 10) of each resin composite were fabricated in a rubber mold with circular cavities (7 mm in diameter and 2 mm thick), in which the composite was inserted in a single increment and compressed with a flat glass slab. The specimens were then light-cured for 20 seconds (Valo Cordless, 1100 mW/cm²; Ultradent, USA) and stored in water for 24 h at 37 °C. The specimen’s surface exposed to the curing light was polished using the Sof-Lex disc system (3M Dental Products, St. Paul, MN, USA) attached to a low-speed handpiece. The discs were used in the grid sequence as follows: coarse; medium; fine; and superfine - 40 seconds each. The initial surface roughness analysis was done with a Surfcorder SE 1700 profilometer (KosakaLab, Tokyo, Japan), considering the Ra parameter. Roughness measurements were taken (reading extension: 2.85 mm; cut-offs: 0.25 mm) three times as the specimen was spun 120° for each taking and a mean value of the three measurements was calculated.

The surface gloss was then measured using a gloss meter (model ZZS 1120, Zehntner Testing Instruments, Switzerland), with the light bean set at the angle of 60° about the specimen’s surface. The gloss meter was connected to a computer and the readings were carried out using a software program (Zehntner gloss tools; 1.0.0014). Four measurements were made for each specimen, rotating the specimen 90° between readings.

The specimens were then submitted to mechanical toothbrushing (MSet; Elquip, Sao Carlos, Sao Paulo, Brazil) using soft-nylon-bristle toothbrushes (Oral-B 35; Gillette do Brasil, São Paulo, SP, Brazil) exerting linear movements at a speed of 200 cycles per minute under a load of 300 g, at 37 °C. Each toothbrush received eight grams of toothpaste (Colgate Total 12; Colgate-Palmolive, São Paulo, SP, Brazil), mixed with 24 mL of distilled water. Surface roughness and gloss were measured before brushing and at 10,000 and 50,000 brushing cycles. Between measurements, specimens were removed from the machine and washed in tap water.

### Statistical Analyses

Data normality was verified using Shapiro-Wilk’s test and the homoscedasticity using Lavene’s test. Statistical analyses were carried out according to the different experimental designs at a significance level of α = 0.05. Data concerning FT, PSS, FLS, FLM, PK (DC and Rp_max_), and GW were statistically analyzed by 2-way ANOVA (factors: resin composite; and preheating and no heating) and Tukey’s test (α = 0.05), using the SPSS software (version 15.0; Statistical Package for the Social Science, SPSS Inc., Chicago, IL, USA). SR and SG data were statistically analyzed using repeated measures ANOVA and the Tukey post hoc test, with two independent variables: the resin composite (FS and UR); and the brushing cycles (0, 10,000, and 50,000) was considered as a sub-parcel.

## Results

Table 1 shows the mean values (µm) for the film thickness (FT) of the resin composites tested. The two-way ANOVA analysis showed a significant difference in thickness concerning the interaction between the two factors assessed (p = 0.046). The preheated groups showed FT mean values significantly lower than those obtained for the non-preheated groups. No statistical difference was observed between UR and FS and between URH and FSH.

**Table 1:** Mean values (µm) and standard deviation of the film thickness considering both heating conditions.

Resin composite	No preheating	Preheating
Universal Restorative	286.83 (16.15) a, A	206.72 (13.88) a, B
Filtek Supreme Ultra	259.60 (26.44) a, A	214.52 (42.85) a, B

* Tukey’s post hoc test: different capital letters in the same row indicate a statistically significant differences. Different lowercase letters in the same column indicate statistically significant differences. Standard deviation in parentheses.

Table 2 shows the mean values and standard deviations for FLS and FLM for both resin composites tested. No statistical difference was observed between UR and FS (FS: p=0.43679; FM: p=0.89089), considering both preheating and non-preheating conditions. Statistical difference (FLS - p=0.01203; FLM - p=0.02079) was observed between preheating and no preheating concerning both materials, where URH and FSH showed higher FLS and FLM mean values than those obtained for UR and FS.

Table 3 shows the PSS mean values and SD for the resin composites tested considering both compliances (Classes I and II) and non- and preheating conditions. No significant difference was observed among the groups concerning both compliances (Class I: p = 0.5030; Class II: p = 0.9270).

Table 4 shows the mean values (µm) for gap width (GW) concerning the resin composites tested. The two-way ANOVA analysis showed no statistical difference between UR and FS (p = 0.4180), considering the non- and preheating conditions. Statistical difference (p = 0.0021) was observed between non- and preheating conditions concerning both UR and FS.

**Table 2:** Flexural strength (FLS) and flexural modulus (FLM) mean values (standard deviation) for resin composites considering both heating conditions.

	FLS (MPa)		FLM (GPa)	
	No preheating	Preheating	No preheating	Preheating
Universal Restorative	141.3 (12.7) a, B	157.9 (2.90) a, A	11.4 (1.2) a, B	13.7 (0.8) a, A
Filtek Supreme Ultra	137.1 (2.43) a, B	152.6 (1.97) a, A	12.2 (1.1) a, B	14.1 (0.7) a, A

* Tukey’s post-hoc test: different capital letters in the same row a indicate statistically significant difference. Different lowercase letters in the same column indicate statistically significant differences. Standard deviation in parentheses.

**Table 3:** Mean values (MPa) and SD for the polymerization shrinkage stress (PSS) considering both compliances (Classes I and II) and heating conditions.

	Class I		Class II	
	No preheating	Preheating	No preheating	Preheating
Universal Restorative	5.57 (1.02)	4.18 (1.31)	1.04 (0.18)	0.85 (0.13)
Filtek Supreme Ultra	4.83 (1.15)	4.22 (1.08)	1.00 (0.16)	1.08 (0.17)

Tukey’s post-hoc test: no significant differences between materials and heating conditions. Standard deviation in parentheses.

**Table 4:** Mean values (µm) and standard deviation of the GW considering non- and preheating conditions.

Resin composite	No preheating	Preheating
Universal Restorative	13.17 (1.08) a, A	9.63 (1.70) a, B
Filtek Supreme Ultra	12.25 (3.16) a, A	9.38 (2.23) a, B

* Tukey’s post-hoc test: different capital letters in the same row indicate a statistically significant differences. Different lowercase letters in the same column indicate statistically significant differences. Standard deviation in parentheses.

Table 5 shows the DC mean values for the resin composites tested. UR showed DC mean values significantly higher than those observed for FS, considering both preheating and non-preheating conditions (p = 0.01075). Preheating showed DC mean values significantly higher than those obtained for non-preheating (p = 0.00031) for both resin composites.

**Table 5:** Mean values (%) for degree of conversion.

Groups	No preheating	Preheating
Universal Restorative	64.21 (1.60) a, B	70.08 (1.41) a, A
Filtek Supreme Ultra	54.66 (2.32) b, B	58.13 (1.04) b, A

* Tukey’s post-hoc test: different capital letters in the same row indicate a statistically significant differences. Different lowercase letters in the same column indicate statistically significant differences. Standard deviation in parentheses.

**Figure 2:**
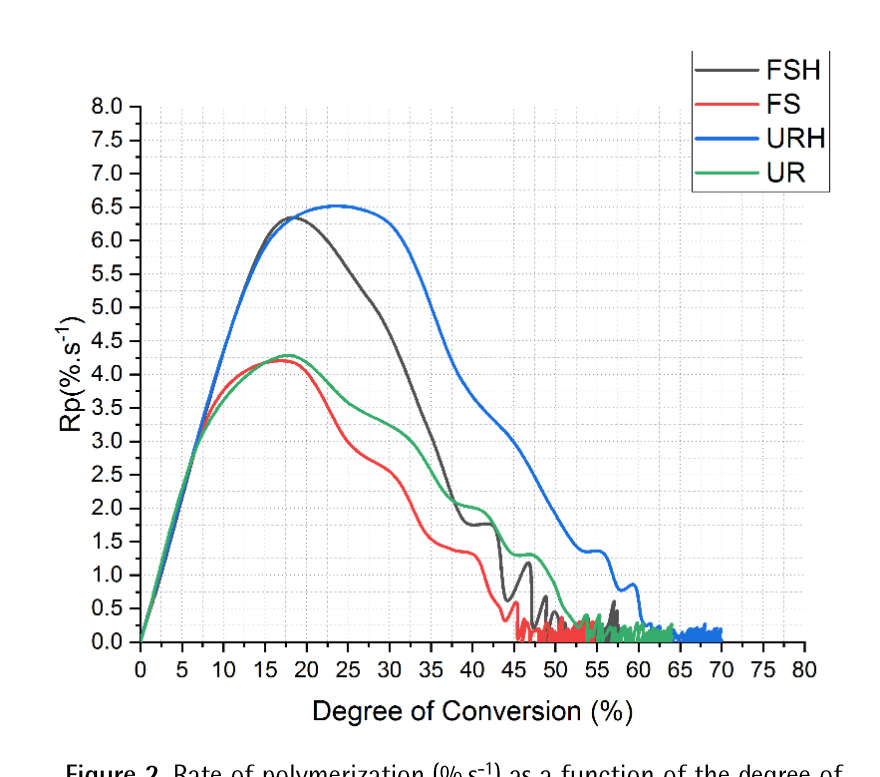
Rate of polymerization (%.s^-1^) as a function of the degree of conversion (%) for the resin composites in non- and preheating conditions. FSH - Filtek Supreme preheated; FS - Filtek Supreme; URH - Filtek Universal Restorative preheated; UR - Filtek Universal Restorative.

Figure 2 shows the polymerization rate (%.s^-1^) as a function of the degree of conversion (%) for the resin composites in non- and preheating conditions considering a photoactivation time range of 300 s. For both resin composites, in the non-preheating condition (FS and UR), the maximum polymerization rate (Rp^max^) reached around 15-17% of the resin composites' degree of conversion, the same occurring with the heated Filtek Supreme composite (FSH), which showed higher Rp^max^ than FS. As for the heated Universal Restorative composite (URH), the Rp^max^ occurred after the degree of conversion reached around 25%.

**Figure 3:**
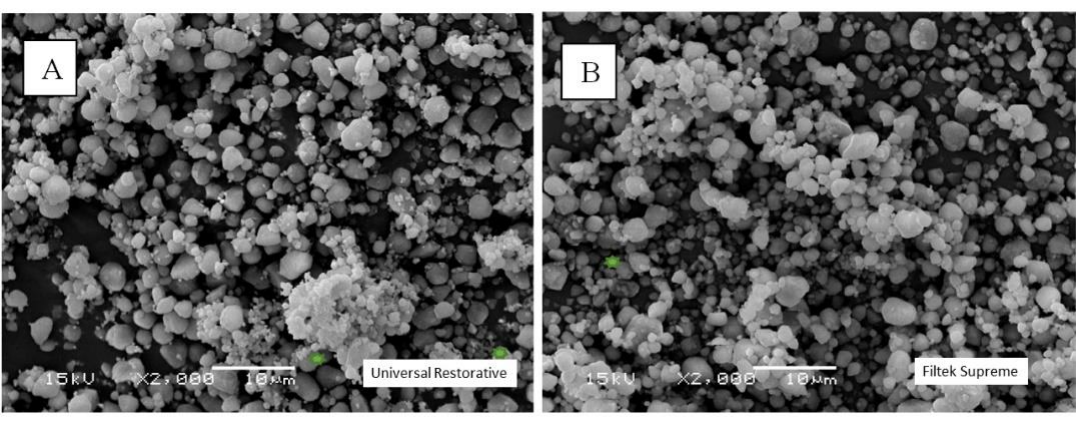
SEM micrographs of the resin composites (×2000): (A) Universal Restorative; and (B) Filtek Supreme. Images show a relatively similar filler particle morphology.

**Figure 4:**
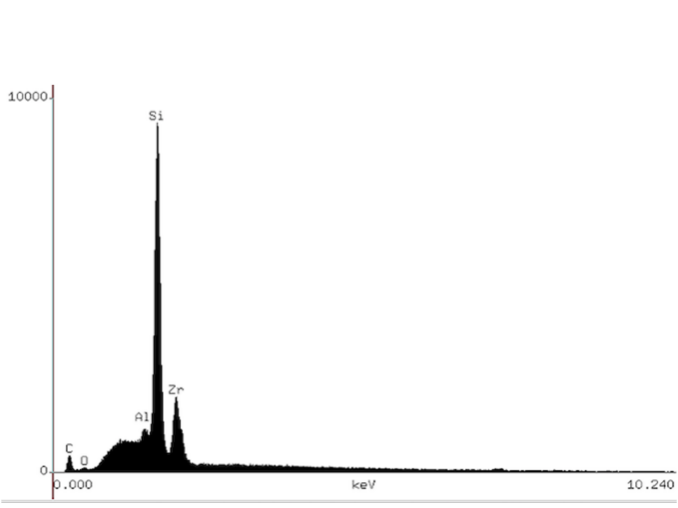
Universal Restorative chemical analysis by X-ray spectroscopy.

**Figure 5:**
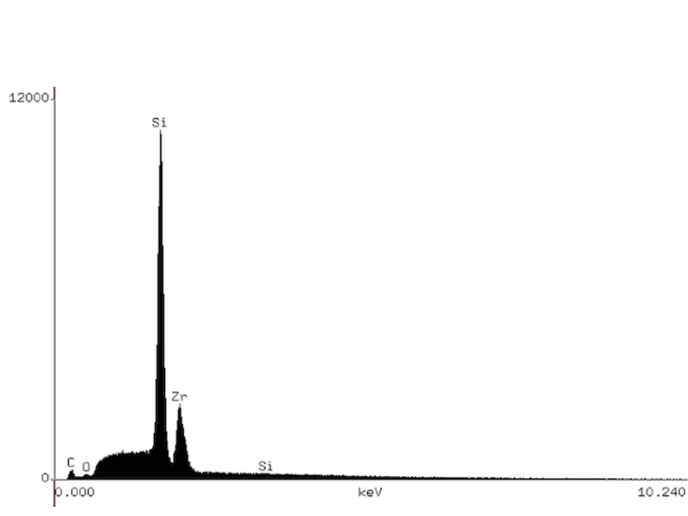
Filtek Supreme chemical analysis by X-ray spectroscopy.

Figure 3 illustrates the SEM images of the filler particles for both resin composites, with the shape and distribution of the particles and nanoclusters being relatively similar. The EDX analysis (Figs. 4 and 5) identified Si and Zr in similar proportions in both resin composites. Al was identified only in Universal Restorative (Figure 4).

**Figure 6:**
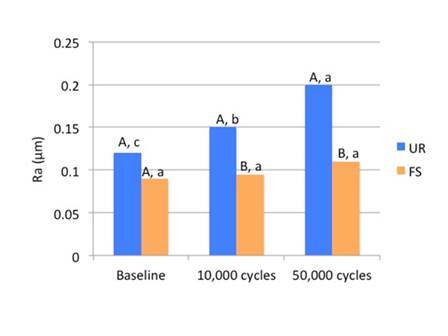
Mean values for surface roughness (Ra, in µm) at baseline and after both brushing cycles. FS - Filtek Supreme; UR - Filtek Universal Restorative. Different capital letters indicate statistical differences between materials. Different lowercase letters indicate statistical differences among brushing cycles.

Figure 6 shows the mean values (Ra in µm) for the surface roughness of the resin composites according to the mechanical brushing cycles (baseline, 10,000 and 50,000). No statistical difference (p = 0.275) in the baseline roughness was observed between FS and UR. After both brushing cycles, UR showed roughness mean values significantly higher than those of FS (p = 0.002). UR roughness was more expressive at 50,000 cycles and statistically different (p = 0.0146) from that recorded at baseline and 10,000 cycles. About the surface roughness of FS, no difference (p = 0.7790) was observed among baseline, 10,000, and 50,000 brushing cycles.

**Figure 7:**
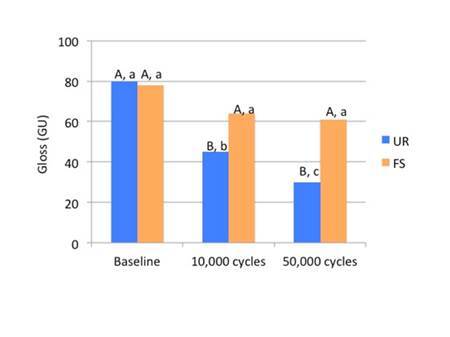
Mean values and standard deviation for surface gloss (GU) after brushing. FS - Filtek Supreme; UR - Filtek Universal Restorative. Different capital letters indicate statistical differences between materials for each brushing cycle. Different lowercase letters indicate statistical differences among brushing cycles.

Figure 7 shows the surface gloss mean values (GU) for the resin composites according to the mechanical brushing cycles (baseline, 10,000 and 50,000). No statistical difference (p = 0.3374) was observed between FS and UR at baseline. After both brushing cycles, FS showed gloss mean values significantly higher than those of UR (p = 0.0014). UR gloss was more expressive at baseline and statistically different (p = 0.0098) from that recorded at 10,000 and 50,000 cycles. No difference (p = 0.8779) in FS gloss was observed among the brushing cycles.

## Discussion

The null hypotheses that (i) the physicochemical and surface properties of the universal resin composite would not significantly differ from those of the conventional composite and that (ii) preheating would have no influence on the physicochemical properties of the resin composites were rejected. Both resin composites and the non- and preheating conditions influenced the properties tested.

When preheated, the two resin composites showed a significant reduction in film thickness (Table 2). The increase in the temperature caused a reduction in their viscosity, allowing a greater flow of the materials under the compressive load ^7^. Despite the reduction in film thickness, the mean values recorded for URH (206.72 µm) and FSH (214.52 µm) exceeded the maximum film thickness value (50 µm) for luting materials according to ISO 4049:2019 specification ^14^, suggesting that preheating is beneficial only for direct restorations.

The greater fluidity observed in the preheated composites might result in better marginal adaptation and reduce the formation of gaps between the restorative material and the tooth (7, 20). The significantly lower gap width mean values obtained for URH and FSH (Table 5), when compared with UR and FS (no preheating), might account for the improved adaptation of the preheated materials in the metallic mold. Because no adhesive system was applied to the cavity walls, these findings reflect only the free volumetric shrinkage of the composite, which is associated with the tension the material exerts on the cavity walls to which it is bonded ^19^. The lower the volumetric shrinkage of the material, the better its adaptation in the dental cavity ^13^.

The PSS mean values of both resin composites in the two simulated compliance conditions for class I and class II cavities were not influenced by non- and preheating conditions (Table 4). Although preheating led to the highest degree of conversion (Table 5) and flexural modulus (Table 3) of both composites, no higher PSS was observed. The increase in monomer conversion occurred due to the greater mobility of the monomers generated by the increase in temperature, which produces a greater free volume in the medium in which the polymerization reaction is taking place, allowing greater movement of the molecules and increasing the number of covalent bonds established between the monomers ^12^. Thus, this finding suggests a possible clinical behavior of the new UR material similar to the favorable results obtained for FS in randomized clinical evaluations ^21^. In addition, preheating increased the maximum polymerization rate (Rp^max^) in both composites (Figure 2) compared to non-preheated composites. Figure 2 also shows that for the Universal Restorative resin composite, heating led not only to an increase in Rp^max^, but also in DC (25%). Although the conversion of monomers into polymers was higher in the preheated groups, this was not enough to have a significant effect on the stress generated in the polymerization ^22^.

The high mean values ​​of DC obtained by the UR resin composite whether preheated or not when compared to the FS resin composite might be explained by the polymeric networks based on urethane monomers, which are more flexible than the Bis-GMA present in the FS. These urethane monomers are capable of rotating the molecular structure ^23^ ^,^ ^24^. Thus, the movement of monomers in later stages of polymerization becomes easier, allowing new bonds to be made in the polymer chains, and increasing the degree of conversion ^24^. On the other hand, the low DC of the FS resin composite might be explained by the presence of Bis-GMA. This monomer has a rigid core molecular structure and establishes strong intermolecular hydrogen bonds ^24^. For this reason, the polymer network formed is less flexible and makes it difficult for monomers to move in advanced stages of polymerization, which impedes new covalent bonds and produces a low degree of conversion ^24^.

The FS and FM mean values for both resin composites increased significantly when they were preheated (Table 3). This result can be explained by the higher DC mean values of the preheated resin composites (Table 5). This possibly occurred because the temperature increased the mobility of the monomers, increasing the number of covalent bonds established between the monomers and also the cross-links between the polymeric chains formed ^12^. Table 3 shows that there was no significant difference between the two resin composites, both in the preheated and non-preheated conditions. Although the two organic matrices are different, the filler particles and the content of these filler particles are similar. Both the type and the content of the inorganic portion in the resin composites are mainly responsible for the mechanical properties of the material ^21^. Box 1 shows that the UR resin composite (76.5% by weight or 58.4% by volume) and the FS (78.5% by weight or 63.3% by volume) have contents close to filler particles and Figure 3 (A and B) show the similar shape and distribution of filler particles in both resin composites. Furthermore, EDS analysis showed that the composition of the filler particles is similar (Figs. 4 and 5). All these findings may have been responsible for the non-statistical difference in the FS and FM of both materials.

Surface analysis was performed without preheating to analyze the morphology and distribution of filler particles and evaluate the performance of these composites under different brushing cycles. The preheating would have little influence on these surface properties, as these properties are more dependent on the filler particles than on the resin matrix, which is the part of the resin composite sensitive to preheating. Regarding these properties, it can be observed that both resin composites did not differ statistically, both in roughness and in gloss baseline, that is, right after the specimens were made (finishing and polishing). However, after periods of 10,000 and 50,000 cycles of mechanical toothbrushing, there was an increase in roughness and a decrease in gloss for the UR resin composite. For the FS resin composite, there was no change in the roughness and gloss mean values when compared to the baseline, both in the periods of 10,000 and 50,000 cycles of mechanical toothbrushing. The literature shows that there is no difference in the roughness and surface gloss of different types of resin composites right after finishing and polishing and they are often the result of the type of abrasive material contained in polishing systems ^25^. In this study, the outcomes obtained at baseline corroborate these findings.

The organic matrix wear may cause the loss of filler particles, exposure of filler particles, matrix cracks, and exposure of trapped air bubbles. These effects are expected after abrasive is wear promoted by mechanical toothbrushing, changing surface roughness, and gloss ^26^. As shown in Box 1 and Figure 3 (A and B) the filler particles and their distribution are similar in both resin composites. They have in common in the inorganic portion, the combination of a non-agglomerated / non-aggregated 20-nm silica filler and a non-agglomerated / non-aggregated 4- to 11-nm zirconia filler. However, UR resin composite also has ytterbium trifluoride fillers consisting of agglomerated 100-nm. An explanation for the higher roughness and lower gloss with the UR resin composite would be that these particles are larger than the others and their exposure on the surface, as well as the voids caused by their detachment during the abrasive process, might have caused greater changes on the surface topography.

UR resin composite showed satisfactory performance when compared to the FS nanoparticulate composite resin and when preheated, reduced the film thickness, gap width, flexural strength, flexural modulus, and increased the DC of the resin composites tested. Thus, this material could be indicated for preheated use in direct restorations. Within the limitations of the present study, controlling the temperature is a great challenge and, technical training of the dental practitioner should be considered to minimize heat loss during handling and application of the material. Further studies considering preheating and other universal resin composites would be relevant to better understand the behavior of these novel types of resin composites. Thus, within the limitations of this study, it is possible to conclude that the universal resin composite tested generally presented similar physicochemical properties compared with the nanofilled resin composite and either similar or slightly inferior surface properties. The preheating improved or maintained all properties evaluated.
